# Applications of Macrocyclic Host Molecules in Immune Modulation and Therapeutic Delivery

**DOI:** 10.3389/fchem.2021.658548

**Published:** 2021-04-06

**Authors:** Shreya S. Soni, Abdulrahman Alsasa, Christopher B. Rodell

**Affiliations:** School of Biomedical Engineering, Science and Health Systems, Drexel University, Philadelphia, PA, United States

**Keywords:** macrocycle, polymer, hydrogel, nanoparticle, drug delivery, immunotherapy, immunology

## Abstract

The immune system plays a central role in the development and progression of human disease. Modulation of the immune response is therefore a critical therapeutic target that enables us to approach some of the most vexing problems in medicine today such as obesity, cancer, viral infection, and autoimmunity. Methods of manipulating the immune system through therapeutic delivery centralize around two common themes: the local delivery of biomaterials to affect the surrounding tissue or the systemic delivery of soluble material systems, often aided by context-specific cell or tissue targeting strategies. In either case, supramolecular interactions enable control of biomaterial composition, structure, and behavior at the molecular-scale; through rational biomaterial design, the realization of next-generation immunotherapeutics and immunotheranostics is therefore made possible. This brief review highlights methods of harnessing macromolecular interaction for immunotherapeutic applications, with an emphasis on modes of drug delivery.

## Introduction

Drug delivery strategies seek to improve therapeutic efficacy by increasing the proportion of drug that reaches its target site (drug targeting), increasing the duration of drug presentation (controlled release), or presenting the drug in response to appropriate triggers (responsive delivery) (Farokhzad and Langer, 2009; Tibbitt et al., 2016; Webber and Langer, 2017). Supramolecular chemistries can advance these goals through the provision of specific, tunable, and thermodynamically reversible bonds. These properties lend themselves to the development of drug delivery systems (Rodell et al., 2015a; Webber and Langer, 2017), especially those that can sequester drugs for subsequent release at the target site, can be tuned to improve pharmacological properties, and can release therapeutic cargo passively or via responsive chemistries.

**Figure 1:**
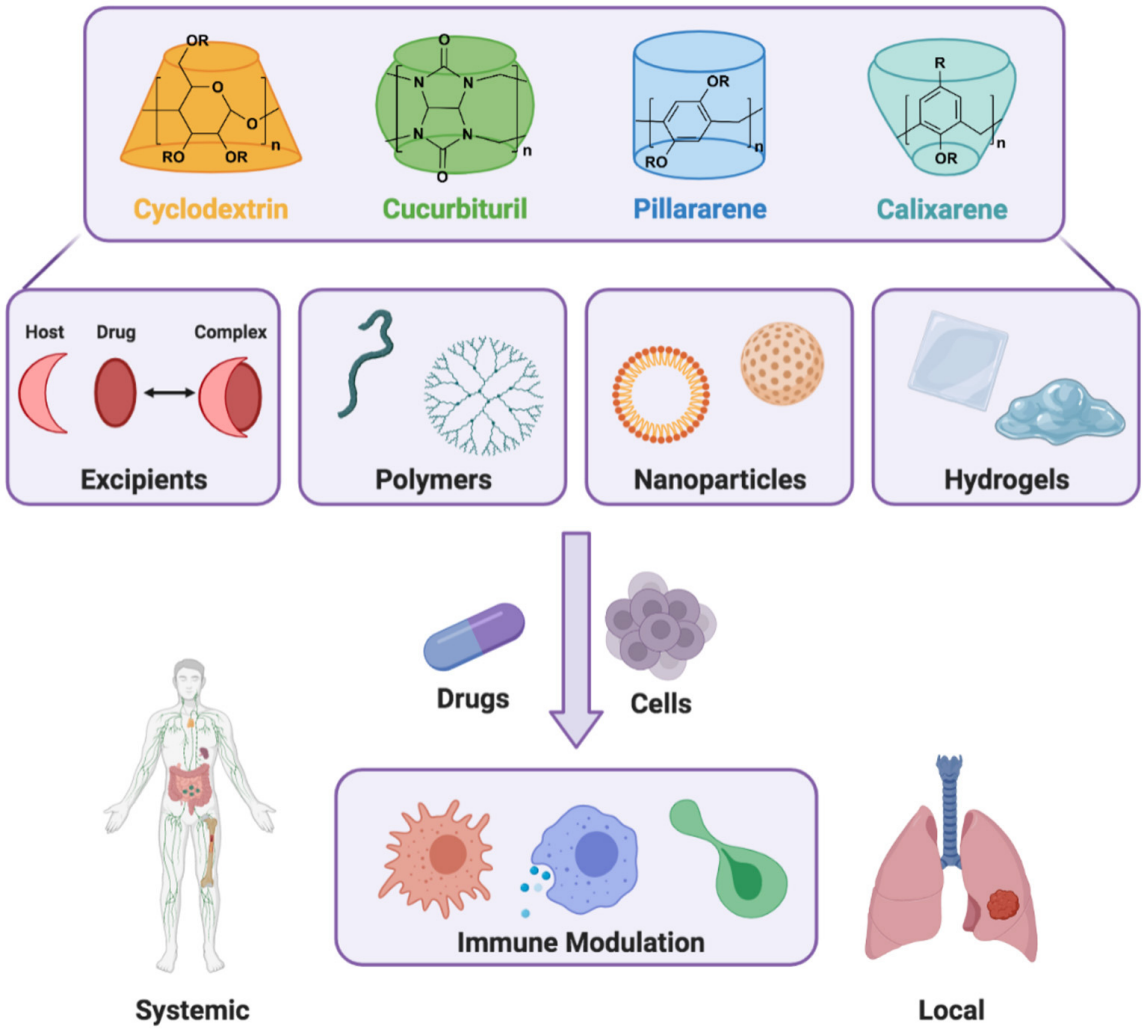
Host macrocycles include the families of cyclodextrins, cucurbiturils, pillararenes, and calixarenes. These molecules contribute to the development of dynamic supramolecular materials, including as formulation excipients and functional building blocks for polymers, nanoparticles, and hydrogels. Through the inclusion of therapeutic cargo (drugs, cells), a range of immunomodulatory outcomes are possible that may span activity throughout the body (systemic effects) or activity at a specific location (local effects), achieved through either targeted systemic delivery or local biomaterial implantation.

Immune system dysfunction is a driver of human disease, for which the delivery of biologic and small molecule drugs to specific tissues and cells is critically needed. Prevalent immune-related diseases include those rooted in non-resolving inflammation, such as cardiovascular disease, arthritis, and tissue injury (Nathan and Ding, 2010). In many such cases, non-specific inflammation manifests in the development of maladaptive autoimmune responses, wherein the body mounts an immune attack against its tissues (Epelman et al., 2015; Jain and Pasare, 2017). Conversely, conditions such as cancer can co-opt the innate immune system to suppress inflammation, thereby thwarting adaptive immunity necessary to combat tumor growth (Engblom et al., 2016). Therapeutic modulation of the immune system is therefore essential and may be used to home in the body's response to various diseases, including mitigation of tissue-damaging inflammation or provocation of an immune response against cancer or infection. To achieve these goals, macrocyclic supramolecules are foundational building blocks that enable drug formulation, cell- and tissue-targeted drug carriers, and local delivery depots to instruct immune cell and systems behavior (Figure 1).

## Macrocycles for Drug Solubilization and Delivery

The development of stable drug formulations and the targeted delivery of therapeutics remain major challenges in pharmacology (Rosenblum et al., 2018; Pasut, 2019; Sanku et al., 2019), and many drugs and drug classes are directly relevant to immune modulation. These include biopharmaceuticals (antibodies, cytokines, chemokines, or peptides) that exhibit biological specificity with well-characterized functions. Many biopharmaceuticals, however, face challenges in formulation stability or rapid blood clearance (Shire et al., 2004; Veronese and Pasut, 2005), and exhibit limited interaction with host macrocycles due to large size and aqueous solubility. Alternatively, small molecule pharmaceuticals are more amenable to guest-host interaction and benefit directly from improved solubility, shielding from degradation, and altered bioavailability. These same supramolecular interactions may also be used to reduce the toxicity of poisonous compounds, either through sequestration or improved clearance (Yin et al., 2021). Macrocycles aid in these processes by acting as hosts to small molecules, imparting aqueous solubility and altered pharmacokinetics by properties inherent to the host molecule structure, through selective chemical functionalization, or by accessing higher-order material structures.

Guest-host interactions are a subset of supramolecular associations characterized by the transient complexation of a macrocyclic cavitand (host) with a small molecule (guest) through hydrophobic interaction, often aided by van der Waal's or electrostatic forces. These macrocycles include cyclodextrins (α-, β-, and γ-CD), cucurbit[n]urils (CB[n]), calix[n]arenes, and pillar[n]arenes (Szejtli, 1998; Lagona et al., 2005; Song and Yang, 2014); all of which potentially enhance drug solubility and bioavailability (Brewster and Loftsson, 2007; Carrier et al., 2007; Loftsson and Brewster, 2011; Walker et al., 2011; Zhou et al., 2015). In many cases, chemical modifications are beneficial toward these goals. For example, sulfobutylether-β-cyclodextrin (SBE-β-CD, Captisol®) is a sulfonic acid derivative of β-CD, currently in 13 FDA approved formulations including antibiotics, antifungals, and antivirals (remdesivir; emergency use approval for COVID-19) (Stella and Rajewski, 2020). Studies by Rajewski demonstrated the derivative's ability to improve drug solubility, formulation stability, and toxicity (Rajewski et al., 1995). Another common CD derivative, 2-hydroxypropyl-β-cyclodextrin (HP-β-CD), possesses excellent water solubility and an improved affinity toward guests such as anti-inflammatory flavonoids, polyphenols, and other compounds (Gould and Scott, 2005; D'Aria et al., 2017; dos Santos Lima et al., 2019). In comparison to CDs, CBs can confer higher affinity interactions that contribute to their utility as potential pharmaceutical excipients, but use may currently be limited by cost and sparing water solubility (Walker et al., 2011; Kuok et al., 2017). Calix[n]arenes and pillar[n]arenes are emergent, synthetically flexible platforms from which a toolbox of drug delivery vehicles is emerging (Zhou et al., 2015; Xiao et al., 2019a,c; Xue et al., 2020).

Macrocyclic delivery vehicles can improve drug pharmacokinetics, including through improved drug solubility (Brewster and Loftsson, 2007; Loftsson and Brewster, 2011) or via receptor-mediated targeted delivery. At the most rudimentary level, macrocycles themselves can serve as targeting agents. CD or its mannose conjugated derivatives are internalized by macrophages and dendritic cells via recognition by cell surface receptors, scavenger receptor A1 (SR-A1) and mannose receptor (MRC1) (Chao et al., 2012; Pustylnikov et al., 2014). For example, mannose-modified β-CD served as a vehicle for the delivery of molecular chaperones to the cytoplasm of macrophages to correct protein misfolding (Rodríguez-Lavado et al., 2014). Similarly, CD-based nanoparticles (NPs) exhibit macrophage uptake, used to achieve trafficking of NPs into glioma (Alizadeh et al., 2010) and the delivery of encapsulated immune agonists to tumor-associated macrophages (TAMs) for cancer immunotherapy (Rodell et al., 2018). Mannose and folate are well-recognized targeting agents for anti-inflammatory (M2-like) and pro-inflammatory (M1-like) macrophages (Ngambenjawong et al., 2017; Rodell et al., 2019a), respectively, and the modification of macrocycles by these moieties enables both macrophage- and tumor-targeted therapies (Okamatsu et al., 2013; Ye et al., 2016; Elamin et al., 2018; Li et al., 2019).

Interestingly, some macrocycles have been investigated for their ability to directly modulate immune response. HP-β-CD may serve as a functional vaccine adjuvant, reportedly altering human dendritic cell maturation, as indicated by an upregulation of inflammatory cytokines (IL-6, TNF-α), production of costimulatory molecules (MHC, PD-L1/2), and activation of co-cultured T lymphocytes (Kim et al., 2016). CB[7] exhibited similar immunostimulatory properties. When complexed with tuftsin, an immunostimulatory tetrapeptide, the complex induced inflammatory cytokine production (TNF-α, IL-2, and IFN-γ) exceeding that of tuftsin alone in mononuclear cells (Kovalenko et al., 2017). While both of these macrocycles exert adjuvant effects, the exact mechanisms of action remain ambiguous. In contrast to these immunostimulatory effects, Zimmer et al. rationalized that the ability of HP-β-CD to bind cholesterol would reduce cholesterol crystal formations known to stimulate macrophage activation. In atherosclerotic plaques, HP-β-CD increased cholesterol efflux, reducing crystal load, inflammation, and disease progression (Zimmer et al., 2016). Similar effects have been observed in monocytes derived from HIV-positive donors (Matassoli et al., 2018), and the compound has been explored clinically for treatment of Niemann-Pick disease type C1, a neurodegenerative disease characterized by excessive cholesterol and lipid accumulation (Liu, 2012; Ottinger et al., 2014).

## Polymer-Bound Macrocycles

Conventionally, polymer-drug conjugates are formed through the covalent tethering of drugs to a polymer. Owing to their relatively large size, polymers dominate the resultant physiochemical properties, and can therefore improve drug solubility, reduce drug clearance rate, and offer sites for attachment of targeting moieties. A distinguishing property of these systems is the structural diversity, which includes end-modified linear polymers, dendrimeric architectures, or pendant modified polymer systems (Elvira et al., 2005; Larson and Ghandehari, 2012). While supramolecular polymer-drug conjugates parallel this structural diversity, they possess advantageous qualities in terms of biocompatibility, ease of modular assembly, and capacity for dynamic behavior (Das et al., 2019).

Conjugation to PEG (i.e., PEGylation) is common in biopharmaceutical modification (Roberts et al., 2002; Alconcel et al., 2011), used to overcome aggregation and denaturation during storage or rapid blood clearance *in vivo* (JevsìŒEvar et al., 2010; Aggarwal, 2014). The interaction between end-modified CB[7]-PEG and the aromatic amino acid residues of proteins enabled PEGylation without chemical modification, including for anti-CD20 antibodies similar to clinical Rituximab (Webber et al., 2016). For small-molecule delivery applications, end-modification of dendrimeric structures has also been explored, including poly(amidoamine) dendrimers modified by α-, β-, or γ-CD that exhibited selective interaction of CD units with small molecule drugs (Wang et al., 2012). Interchain modifications of PEG may also be useful, as for self-assembly of polymeric NPs. CRLX101 (i.e., IT-101) was formed through covalent conjugation of camptothecin to a linear β-CD-PEG copolymer (Davis, 2009). Due to interaction of camptothecin and β-CD, the polymer chains condensed into NPs that modulated the immune response in tumor-bearing mice, including activation of natural killer cells and T cell proliferation (Chen Y. F. et al., 2019).

The modification of linear polymers by pendant groups has been accomplished through several means. Polyrotaxanes are one such dynamic macromolecular structure, composed of macrocycles threaded along a polymer chain. When modified by targeting ligands, such as maltose or mannose, macrocycles slide along the polymer chain to form multivalent interactions with target receptors, improving molecular recognition and macrophage uptake (Ooya et al., 2003; Shibaguchi et al., 2019). These methods emphasize how the design of dynamic supramolecular structures can improve targeted delivery. In an excellent example of modular conjugation, Jung et al. covalently modified hyaluronic acid with pendent CB[6] groups (Jung et al., 2011). The resulting CB[6]-HA was decorated with FITC for bioimaging and/or a formyl peptide receptor like 1 (FPRL1) peptide ligand with application in leukocyte recruitment, both using spermidine (CB[6] guest) as a supramolecular tether. The multi-functional approach provides a means of modular on-demand assembly that can achieve targeting, bioimaging, and drug delivery in a single supramolecular vehicle; such supramolecular theranostics have been recently reviewed (Yu and Chen, 2019).

## Nanoparticulate Systems

Particulate platforms are among the most explored systemic drug delivery systems, exhibiting similar effects as polymeric drug conjugates: enhanced drug solubility, prolonged blood clearance, and targeted delivery (Mudshinge et al., 2011). Numerous methods have been developed to leverage guest-host interactions in nanotherapeutic design, including the self-assembly of subunits, surface modifications of pre-formed particles, and nanogels for affinity-based delivery.

An excellent application of supramolecular assembly is in gene delivery systems, wherein cationic polymers complex with nucleic acids through electrostatic interactions to form polyplexes, protecting the cargo from enzymatic degradation and facilitating cytoplasmic delivery (Lächelt and Wagner, 2015). While branched polyethyleneimine (PEI) is regarded as the “gold standard” polymeric vehicle, it is limited by cytotoxicity (Godbey et al., 1999; Lungwitz et al., 2005). CD-PEI conjugated systems have therefore been established that decrease the molecular weight of PEI necessary for effective transfection, hence improving cell viability (Wong et al., 2018). Similar methods have been applied to other cationic polymers with comparable limitations, like poly(2-dimethylaminoethyl methacrylate) (PDMAEMA) and dioleoyl-3-trimethylammonium propane (DOTAP) (Cherng et al., 1996; Loh and Wu, 2015; Fan et al., 2018; Zhou et al., 2018). Arima et al. prepared a sugar-appended CD-dendrimer conjugate as a macrophage-targeted carrier for systemic delivery of gene therapies (Arima et al., 2013), while local delivery has been enabled by CD-PEI conjugates for siRNA delivery from injectable hydrogels (Wang et al., 2017). In addition, Chang and colleagues investigated the co-delivery of an anticancer drug and small interfering RNA (siRNA) in a self-assembled redox-responsive pillar[5]arene nanocarrier, designed to overcome chemotherapeutic drug resistance (Chang et al., 2014). In sum, macrocycles are effective in enhancing gene delivery, due to their ability to reduce the cytotoxicity of cationic polymeric vectors, increase membrane permeability, and contribute to targeted or responsive delivery systems. Further developments in these areas have been recently reviewed (Xiao et al., 2019a; Haley et al., 2020).

Particulate materials are likewise of utility in vaccine delivery, aided by modular supramolecular assembly. In anti-tumor applications, the self-assembly of a MUC1 vaccine nanoparticle was achieved through CB[8] linkage of an amphiphilic TLR2 agonist (Pam_3_CSK_4_) with the desired antigen. Resulting nanostructures triggered a more robust immune response in mice than soluble controls (Gao et al., 2014). Interestingly, macrocycles may also serve to open new avenues for orally administered vaccines. He et al. encapsulated an ovalbumin β-CD complex in chitosan NPs. Oral administration to mice increased antibody levels in the digestive tract mucosa and serum, demonstrating the nanostructure's ability to induce an adaptive immune response (He et al., 2019). Anti-inflammatory regulation of the digestive tract has also been achieved, including by the intravenous administration of rosiglitazone-loaded redox-responsive nanoparticles that modulated macrophage response in ulcerative colitis (Sun et al., 2020).

Hydrogel NPs, or nanogels, are composed of cross-linked polymeric networks that provide a large surface area for multivalent supramolecular conjugation, making them excellent materials for drug loading, targeting, and release (Oh et al., 2008; Suhail et al., 2019). Park and colleagues formulated liposomal polymeric gels, composed of β-CD (for conjugation of a TGF-inhibitor) and a polymeric network (for IL-2 encapsulation) that significantly delayed tumor growth by synergistically activating the innate and adaptive immune response, increasing survival of tumor-bearing mice (Park J. et al., 2012). More recently, cyclodextrin nanoparticles were prepared by crosslinking succinylated β-CD with *L*-lysine. Resulting NPs exhibited uptake by myeloid cells (macrophages, dendritic cells) for the targeted delivery of immunostimulatory drugs, including TLR7/8 agonists (Kim et al., 2018; Rodell et al., 2018, 2019b), non-canonical NF-κB activators (Koch et al., 2020), and other drugs (Ahmed et al., 2019). These applications have demonstrated improved targeting of drugs to macrophage-rich tissues and a concurrent reduction in off-target drug effects, particularly for drugs with high-affinity interactions.

## Bulk Materials for Localized Action

Complementary to systemic drug delivery by soluble polymeric and nanomaterial systems, macroscale biomaterials provide an opportunity for highly localized therapeutic delivery. Local therapy poses potential advantages (Weiser and Saltzman, 2014), including a reduction in off-target side effects such as adverse immune suppression or activation, which place patients at risk for infection or hyperinflammatory conditions (cytokine release syndrome), respectively. Related applications encompass device coatings, implantable delivery depots, and injectable hydrogels that leverage supramolecular guest-host interactions either for therapeutic drug sequestration or hydrogel crosslinking.

Medical device implantation is commonplace in modern medicine, such as for diagnostic or reconstructive procedures. Yet, these devices are hampered by biofilm formation: the colonization of the implant surface by bacteria or fungi (Arciola et al., 2018). To impede biofilm formation, antifouling and/or drug-eluting surfaces are of great interest. Covalent tethering of CD to implant surfaces (El Ghoul et al., 2008; Nava-Ortiz et al., 2010), as well as polymerized CD coatings have been investigated (Learn et al., 2020). These methods generally reduced protein adsorption and cell adhesion. Moreover, they provided affinity-based release of antibiotic and antifungal drugs, thereby inhibiting biofilm formation (El Ghoul et al., 2008; Nava-Ortiz et al., 2010; Thatiparti and Von Recum, 2010; Learn et al., 2020). In sum, surface modification by macrocycles is a promising method for the provision of antifouling surfaces that allows for biofilm inhibition by local prophylactic drug delivery.

**Figure 2:**
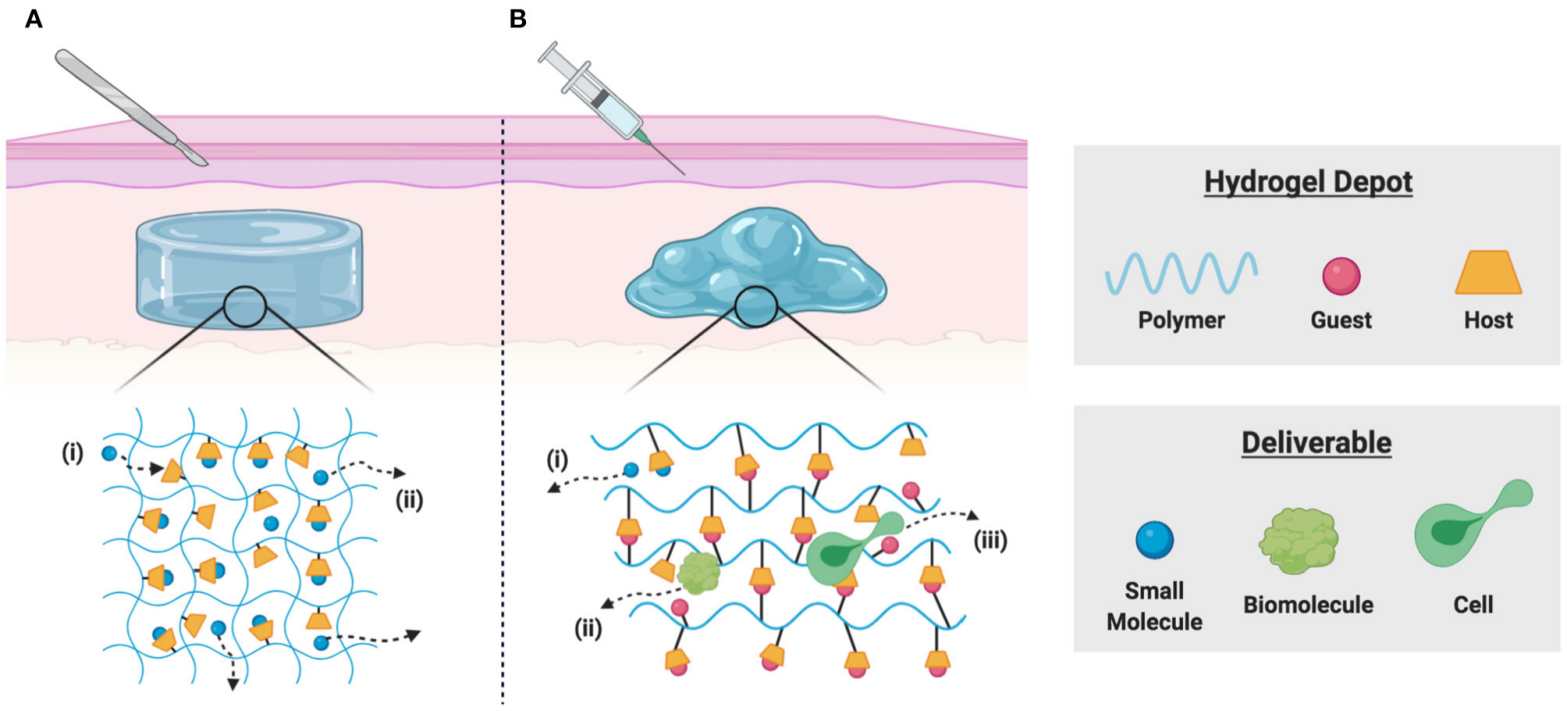
Schematic representation of local delivery from implantable and injectable hydrogels. **(A)** Polymer-bound macrocycles form inclusion complexes with desired deliverables in implantable hydrogels, allowing for (i) sequestration of drugs through guest-host complexation and (ii) affinity-based release by diffusion of unbound drug molecules. **(B)** Polymer-bound host macrocycles can form reversible physical bonds with hydrophobic guest molecules, allowing for the formulation of dynamic supramolecular crosslinks. These shear-thinning and injectable materials enable (i) drug sequestration through guest-host affinity, (ii) diffusive release of soluble biomolecules, and (iii) dynamic bond rearrangement necessary for cell migration into or out of the scaffold.

To achieve local delivery within tissues, the formation of hydrogel depots is widely used for various applications. These water-swollen polymer networks enable controlled release via diffusive, degradation-mediated, or externally triggered mechanisms (Li and Mooney, 2016). Small molecule drugs, however, exhibit rapid diffusive release due to the relatively large mesh size (Bertz et al., 2013), motivating the development of hydrogels which include macrocyclic supramolecules for affinity-based delivery (Figure 2A). Partially crosslinked CDs and CD-modified gellan gum have been used to develop injectable polymers useful as viscosupplements with concurrent intraarticular release of anti-inflammatory glucocorticoids with applications in osteoarthritis treatment (Rivera-Delgado et al., 2018; Choi et al., 2020). Researchers have used these hydrogel formulations to deliver cyclosporin A (CsA), an immunosuppressant used to prevent organ transplant rejection and whose systemic administration is limited by off-target effects (Liddicoat and Lavelle, 2019; Park et al., 2020). Hydrogels prepared from poly(HEMA-co-HP-β-CD) provided controlled release of CsA over 2 months *in vitro*, with potential applications in subjunctival delivery following corneal graft procedures (Başbag et al., 2014). For the promotion of burn wound healing, dual delivery strategies have been described, including *in situ* polymerizable hydrogels for affinity-based release of resveratrol (an anti-inflammatory) and a plasmid encoding vascular endothelial growth factor (to promote vascularization) (Wang et al., 2019). Co-delivery of resveratrol and histatin-1 has been similarly demonstrated, using co-polymerization of acrylated β-CD and methacrylated gelatin for controlled release (Zheng et al., 2020).

Injectable hydrogel formulations are a convenient means of delivery, requiring less invasive procedures for implantation than solid hydrogels formed *ex vivo* (Yu and Ding, 2008). Injectable hydrogels may be delivered in a liquid state, later solidifying as a result of thermo-responsive condensation or external triggers (Nguyen and Lee, 2010). In contrast, supramolecular assembly enables the formation of shear-thinning, injectable hydrogels (Guvendiren et al., 2012; Rodell et al., 2015a). The hydrogels can be pre-formed in a syringe with encapsulated therapeutics, injected into the tissue, and re-form as a depot for subsequent therapeutic release. One avenue for hydrogel formation is through pseudo-polyrotaxane formation between α-CD and PEG (Li et al., 1994). These hydrogels have found recent use in cancer immunotherapies, where the constraint of immune activation to the tumor environment is desirable to prevent systemic toxicity. Wang and colleagues used a hydrogel composed of α-CD and 4-arm PEG for local delivery of an adenoviral vector encoding Flagrp170, a flagellin-derived NF-κB stimulating sequence shown to enhance tumor immunogenicity (Wang et al., 2020). In a murine melanoma model, the hydrogel improved vector retention at the tumor site, facilitating local immune activation and suppression of tumor growth. CDs have also been recently used to create a hydrogel-particle composite for synergistic photothermal immunotherapy. In this work, IR820-α-CD was used in hydrogel formation, and localized heating by IR light induced tumor cell death; embedded CpG nanoparticles supported the immunotherapeutic effect of tumor-derived antigens (Dong et al., 2019).

The modification of polymers by pendant guest and host groups is an alternative means of constructing supramolecular hydrogels (Rodell et al., 2015a; Xiao et al., 2019b). Hydrogels crosslinked by β-CD and adamantane interaction have been widely investigated as injectable therapeutics (Loebel et al., 2017), including for the delivery of small-molecule drugs (Mealy et al., 2015; Zheng et al., 2020), biomolecules (Rodell et al., 2013, 2015b; Soranno et al., 2016), extracellular vesicles (Chen et al., 2018; Chung et al., 2020), and cells (Gaffey et al., 2015, 2019; Sisso et al., 2020) (Figure 2B). The delivery of IL-10 has been demonstrated, both from these guest-host hydrogels in the injured kidney (Rodell et al., 2015b; Soranno et al., 2016), and from related supramolecular hydrogel/microgel composites in the infarcted heart (Chen M. H. et al., 2019) as a means of promoting tissue healing. Similar guest-host hydrogels may be formed through the association of CB[n] hosts with polymer-bound guests (Appel et al., 2010), which has been used to improve local cell retention and in the development of numerous responsive drug delivery systems (Park K. et al., 2012; Ding et al., 2019). In an interesting application, guest-host hydrogels were prepared from the interaction of gelatin with photocrosslinkable acrylated β-CD as a 3D co-culture platform for TAM repolarization. IFN-γ reverted macrophages to a pro-inflammatory phenotype *in vitro* and decreased tumor cell migration and proliferation. Hydrogels were readily disassembled by competitive binding of free adamantane, allowing co-cultured cells to be transplanted into tumor growth models *in vivo*, where TAM repolarization inhibited tumor growth (Huang et al., 2020). Such platforms are a valuable drug discovery tool, and such accessible platforms for 3D cell culture are highly desirable (Caliari and Burdick, 2016; Rodell et al., 2019a). These self-assembling systems may furthermore be useful for immune modulation *in vivo*. For example, Widener et al. recently reported the preparation of granular hydrogel assemblies, wherein microgels were separately modified by β-CD or adamantane groups to yield self-assembling and injectable granular assemblies (Widener et al., 2020). The highly interconnected pores between the microgels allowed rapid immune cell migration and may provide an excellent platform for cellular reprogramming. Indeed, related polymer-nanoparticle composites enable recruitment and differentiation of discrete cell subsets (Fenton et al., 2019), and microgel architecture itself can promote distinct changes in the secretory profile of cells upon their arrival (Caldwell et al., 2020).

## Conclusion

Supramolecular chemistry has emerged as a new frontier for biomedicine, providing a synthetically tractable route to the design of dynamic supramolecular, macromolecular, and multiscale material systems. By appropriate use of the custom design of macrocyclic building blocks, influence over material properties and biological outcomes is made possible. In immune engineering, these tools uniquely enable access to the same thermodynamic principles that underly biological structures, which has rapidly led to the development of methods to overcome previously insurmountable pharmacological obstacles, including drug solubility and instability or roadblocks to physiological transport. Moreover, the expanding toolbox of macrocyclic biomaterials now accessing multifunctional materials, such as immunotheranostics that perpetuate the combined study of vehicle and drug pharmacokinetics alongside pharmacodynamic outcomes. Such platforms will allow the unification of cell and tissue level response with vehicle and drug biodistributions, which previously have been difficult to access (Rodell et al., 2020). Moreover, the modularity of macrocyclic interactions perpetuates the development of delivery systems with tunable drug compositions. Importantly, cargo sequestration typically requires no chemical modification and therefore forgoes the formation of new chemical entities. In contrast, the addition of well-understood guest anchors to existing drugs also allows for tunable drug affinities for applications in controlled release, responsive delivery, and *in situ* refillable drug reservoirs (Rodell et al., 2019b; Zou et al., 2019; Dogan and von Recum, 2020; Dogan et al., 2020). Looking forward, these tools may be leveraged to directly address shortcomings in therapeutic efficacy, off-target drug effects, and dosing frequency that hamper the success of immunotherapeutic drugs in practice.

## Author Contributions

SS and AA conducted the initial primary literature review, and all authors contributed to the writing and editing of the final manuscript.

## Conflict of Interest

The authors declare that the research was conducted in the absence of any commercial or financial relationships that could be construed as a potential conflict of interest.
